# ACE2 alleviates sepsis-induced cardiomyopathy through inhibiting M1 macrophage via NF-κB/STAT1 signals

**DOI:** 10.1007/s10565-024-09923-z

**Published:** 2024-09-25

**Authors:** Xue Xiao, Jia-Xin Li, Hui-Hua Li, Fei Teng

**Affiliations:** Department of Emergency Medicine, Beijing Key Laboratory of Cardiopulmonary Cerebral Resuscitation, Beijing Chao-Yang Hospital, Capital Medical University, No. 8 Worker’s Stadium South Roud, Beijing, 100020 China

**Keywords:** ACE2, Sepsis-induced cardiomyopathy, Bone marrow transplantation, Macrophage polarization

## Abstract

Angiotensin-converting enzyme 2 (ACE2), a crucial element of the renin-angiotensin system (RAS), metabolizes angiotensin II into Ang (1–7), which then combines with the Mas receptor (MasR) to fulfill its protective role in various diseases. Nevertheless, the involvement of ACE2 in sepsis-induced cardiomyopathy (SIC) is still unexplored. In this study, our results revealed that CLP surgery dramatically impaired cardiac function accompanied with disruption of the balance between ACE2-Ang (1–7) and ACE-Ang II axis in septic heart tissues. Moreover, ACE2 knockin markedly alleviated sepsis induced RAS disorder, cardiac dysfunction and improved survival rate in mice, while ACE2 knockout significantly exacerbates these outcomes. Adoptive transfer of bone marrow cells and *in vitro* experiments showed the positive role of myeloid ACE2 by mitigating oxidative stress, inflammatory response, macrophage polarization and cardiomyocyte apoptosis by blocking NF-κB and STAT1 signals. However, the beneficial impacts were nullified by MasR antagonist A779. Collectively, these findings showed that ACE2 alleviated SIC by inhibiting M1 macrophage via activating the Ang (1–7)-MasR axis, highlight that ACE2 might be a promising target for the management of sepsis and SIC patients.

**Supplementary Information:**

The online version contains supplementary material available at 10.1007/s10565-024-09923-z.

## Introduction

Sepsis is defined by multi-organ dysfunction due to a pathological host response to infections and causes severe adverse outcomes (Cecconi et al. 2018). During sepsis, the innate immune system acts as the primary defense for the host, with immune cells like monocytes/macrophages becoming hyperactivated, releasing copious inflammatory cytokines, such as interleukin (IL)-1β, IL-6 and interferon-gamma (IFN-γ). This phenomenon, known as a cytokine storm, leads to multiple organ dysfunction (Zhang et al. 2023b). The heart is a primary target of the inflammatory response, and sepsis-induced cardiomyopathy (SIC) is frequently diagnosed when functional disorders occur in the heart during sepsis, including systolic dysfunction and perfusion failure (Ehrman et al. 2018; Hollenberg and Singer 2021; Liu et al. 2023). In patients with SIC, the mortality rate is elevated by 2 to 3 times (Ehrman et al. 2018) and the mechanisms of SIC involve multiple pathways. For instance, imbalance of pro- and anti-inflammatory cytokine expression, release of nitric oxide and reactive oxygen species (ROS), downregulation of sarcomere and mitochondrial proteins, coronary microvascular disturbance, and abnormal calcium processing have been observed (Hollenberg and Singer 2021). However, the treatment and prognosis of SIC patients remain uncertain.

ACE2 is essential in balancing the adverse effects of ACE within the renin-angiotensin system (RAS) (Tipnis et al. 2000). ACE and ACE2 exhibit significant homology but have opposing effects. ACE facilitates the transformation of angiotensin (Ang) I into the Ang II, which can activate the angiotensin II type 1 receptor (AT1R) for various biological functions. Conversely, ACE2 converts Ang II to the vasodilator Ang (1–7) and binds to the Mas receptor (MasR) to exert anti-inflammation and cell protective effect (Karki et al. 2021). Intriguingly, mounting evidence highlights the connection between the RAS and cardiovascular dysfunction, such as atherosclerosis, hypertension, heart failure, and myocardial infarction, as well as even sepsis and SIC (Bai et al. 2022; Xu et al. 2021). Previous research reported that male C57BL/6 ACE2 knockout mice exhibit normal cardiac systolic function and mild elevation of systolic blood pressure (SBP) compared to their WT mice at 6 to 12 months of age (Gurley et al. 2006). One study indicates that ACE2 knockout mice under 3 months of age had no significant difference in body weight and blood glucose compared to WT mice (Wong et al. 2007). Our recent findings and other study also confirmed that 8–10-week-old mice with either overexpression or deletion of ACE2 developed normally compared to wild-type animals, which show no detectable abnormalities in blood pressure, body weight, liver function (measured by serum aspartate transaminase and alanine transaminase) and renal function (assessed by serum creatinine and creatinine clearance) (Li et al. 2024; Liu et al. 2024; Ni et al. 2020), suggesting that ACE2 overexpression or deletion has no significant effects on multiorgan function under baseline. However, under disease conditions, ACE2 overexpression exhibits protective effects. For example, ACE2-Ang (1–7)-MasR signal exhibits a beneficial effect in the regulation of cardiovascular remodeling and dysfunction (Xu et al. 2021). Ang (1–7) prevents sepsis-induced left ventricular (LV) damage caused by lipopolysaccharide (LPS) (Xu et al. 2021). ACE2 also mitigates inflammation through modulating M1/M2 macrophage polarization via the MAPK and NF-κB signals (Chen et al. 2023; Xu et al. 2021). Nevertheless, the molecular mechanisms of this axis in SIC remain largely elusive.

The current study demonstrated that ACE2 exerts a protective effect in SIC after cecal ligation and puncture (CLP) surgery. Our findings indicated that the CLP-induced sepsis resulted in a disturbance characterized by the enhancement of the ACE-Ang II-AT1R axis and the suppression of the ACE2-Ang (1–7)-MasR pathway. ACE2 overexpression in mice remarkably improved survival rate and cardiac damage, reduced the production of Ang II and ROS along with M1 macrophage polarization while increased the generation of Ang (1–7) in mice post-CLP surgery. Conversely, ACE2 knockout had the opposite effects. All findings were confirmed by bone-marrow (BM) transplantation experiments. Mechanistically, ACE2 overexpression in myeloid cells alleviated SIC by activating the Ang (1–7)-MasR-regulated inhibition of NF–κB/STAT1 signals. Taken together, this research suggests that enhancing ACE2 activity could be a promising therapeutic approach for sepsis and SIC.

## Materials and methods

### Animals

Global ACE2 knockout (KO; KOCMP-70008-ACE2-B6J-VA) and knockin (KI; KIAIAS200927WZ2-B) mice were generated by Cyagen Biosciences (Guangzhou, China) on the C57BL/6 J strain and were identified as reported previously (Liu et al. 2019). WT mice (C57BL/6 J) were obtained from the Cyagen Biosciences. The mice were kept and reproduced in a facility that was free of specific pathogens, following standard housing conditions that included 12 h of light and darkness. All experiments were conducted according to the Declaration of Helsinki and the NIH Guidelines for the Care and Use of Laboratory Animals, and the ethics approval for the current research was granted by the Ethics Committee at Beijing Chao-Yang Hospital.

### Model of polymicrobial sepsis and SIC

Murine polymicrobial sepsis model was created by CLP in mice aged 8 to 10 weeks as reported previously (Zhang et al. 2023a). In brief, animals were anesthetized by inhalation of 2–3% isoflurane and underwent a 1 cm midline laparotomy procedure in the CLP group. Then, the cecum was ligated and punctured using a 21-gauge needle. In the sham group, same procedure was conducted without puncture*.* 24 to 72 h later, all animals were deeply anesthetized with 4–5% isoflurane inhalation and euthanized by cervical dislocation and bloodletting at 24, 48 or 72 h after the CLP operation. Blood and heart tissues were collected for further study.

### Blood pressure monitoring

Blood pressure of all mice was monitored non-invasively utilizing the tail-cuff method (softron BP-2010A) as reported previously (Wang et al. 2016). Gently place the mouse into the holder heated to 36℃ for 5 min to maintain stability. Then, perform noninvasive blood pressure measurements at one-minute intervals and record the data.

### Echocardiography

Echocardiography was used to evaluate cardiac function with a Vero 2100 High-Resolution Imaging System as reported previously (Feng et al. 2023). Animals were given anesthesia using 1.5% isoflurane. Echocardiographic parameters, including left ventricular wall thickness, LV chamber and internal dimensions were obtained from original M-mode tracings over three cardiac cycles. Calculations were performed for the percentages of ejection fraction (EF%) and fractional shortening (FS%) of the LV as reported.

### Biochemical measurements

The levels of lactate dehydrogenase (LDH, indicator of heart function) (A020-1–2, Nanjing Jiancheng, China) and ACE2 activity (P0319S, Beyotime, China) in plasma and cardiac tissues were detected according to the manufacturer’s guidelines. The heart tissues were cut and homogenized by an ultrasonic processor in lysis buffer, and the concentrations of total protein were assessed. The concentrations of Ang II, ACE and Ang (1–7) in mouse heart lysates and plasma were detected using ELISA kits (SEA004Mu, CEA005Mu, CES085Mi, Cloud-Clone Corp, China).

### Myocardial histopathology

Heart tissues were preserved in 4% paraformaldehyde and embedded in paraffin. Immunohistochemistry was performed using anti-Mac-2 (ARG66239, 1:200; Arigo) antibodies. Another part of the heart was embedded in OCT (Sakura Finetek) to obtain cryosections and then stained with dihydroethidium (DHE, 1 µmol/L) to analyze the superoxide contents. Fluorescence images was captured from 6 randomly selected fields for each sample using a Nikon microscope (Nikon, Tokyo, Japan).

### Bone marrow (BM) transplantation

BM transplantation was conducted according to the protocols previously outlined (Wang et al. 2018). Briefly, 8-week-old male WT mice were selected as recipients and exposed to a lethal dose of radiation (8.5 Gy). BM cells were isolated from tibia and femur of WT, ACE2-KI and ACE2-KO mice with RPMI-1640 medium. Each recipient WT mouse received 1–2 × 10^7^ BM cells. After 8-week recovery period, all mice were exposed to CLP for 24 h.

### Western blotting

Total protein samples were purified from fresh heart tissues or cells utilizing radioimmunoprecipitation assay lysis buffer and quantified with a protein assay kit (Pierce Chemical). Western blot analysis was performed as descripted (Wang et al. 2016) using anti-ACE (ARG41098, Arigo), anti-ACE2 (ab183506, Abcam), anti-AT1R (25343–1-AP, Proteintech), anti-MasR (20080–1-AP, Proteintech), anti-p65-NF-kB (8242S, CST), anti-P-p65-NF-kB (3033S, CST), anti-STAT1 (14995S, CST), anti-P-STAT1 (19167S, CST) anti-GAPDH (5174S, CST), and anti-β-actin (4970S, CST). Quantification was performed with ImageJ software.

### Quantitative PCR (qPCR) analysis

Total RNA samples were purified from fresh heart tissues and cells with the TRIzol reagent kit (9109, Takara). The qPCR was conducted on an iCycler IQ system as reported (Wang et al. 2018). The primers were used for assessing the mRNA levels of the CD80, IL-1β, IL-6, TNF-α, CD206, IL-10, arginase-1 (ARG-1), nicotinamide adenine dinucleotide phosphate oxidase (NOX2), Bax and Bcl-2. All primers were obtained from Sangon Biotech (Shanghai, China). All values were quantitated using the 2^−ΔΔCT^ method and normalized to internal controls (GAPDH or β-actin). Supplementary Table S1 contains a list of the primers.

GAPDH, Glyceraldehyde 3-phosphate dehydrogenase; IL-1β, Interleukin-1β; IL-6, Interleukin-6; TNF-α, Tumor necrosis factor; NOX2, nicotinamide adenine dinucleotide phosphate oxidase 2; IL-10, Interleukin-10; ARG-1, arginase-1; Bcl-2, B-cell lymphoma 2 and Bax, BCL2-associated X protein.

### Co-culture of neonatal rat cardiomyocytes (NRCMs) and bone marrow-derived macrophages (BMDMs)

BMDMs were extracted from WT and ACE2-KI mice as reported previously (Wang et al. 2018). After cervical dislocation the femurs and tibias were flushed using a 1 ml syringe with RPMI-1640 medium. The BM cells were incubated in RPMI-1640 medium containing 10% FBS, and subsequently stimulated with 20 ng/ml M-CSF (#315–02, PEPROTECH) for 72 h to promote monocyte differentiation into M0 macrophages. Macrophages were activated using 1 ng/μL LPS (L2637, Sigma, Germany) or saline for 6 h. NRCMs were extracted from neonatal Sprague–Dawley rats. The heart tissues were excised and cut into pieces and subjected to digestion using 0.04% trypsin and 0.07% type II collagenase as reported previously (Xie et al. 2018). The cardiomyocytes were then moved to the upper chamber of a 6-well Transwell insert, while macrophages were positioned in the lower chamber and treated with A779 (10^–5^ mol/L) (HY-P0216, MCE) and/or LPS (1 ng/μL) for an additional 24 h.

### Statistical analysis

All results were represented as the mean ± SEM and analyzed with Prism software 9.0. For normal distributed results, comparation between two groups was performed with unpaired t test, while nonparametric data were assessed by the Mann–Whitney test. One-way or two-way ANOVA analysis was used for comparations of multiple groups. The log-rank (Mantel‒Cox) tests were selected for determining the differences in the mouse survival rate. A *P* value below 0.05 was deemed statistically significant.

## Results

### RAS balance of the heart is disturbed by CLP

**Fig. 1:**
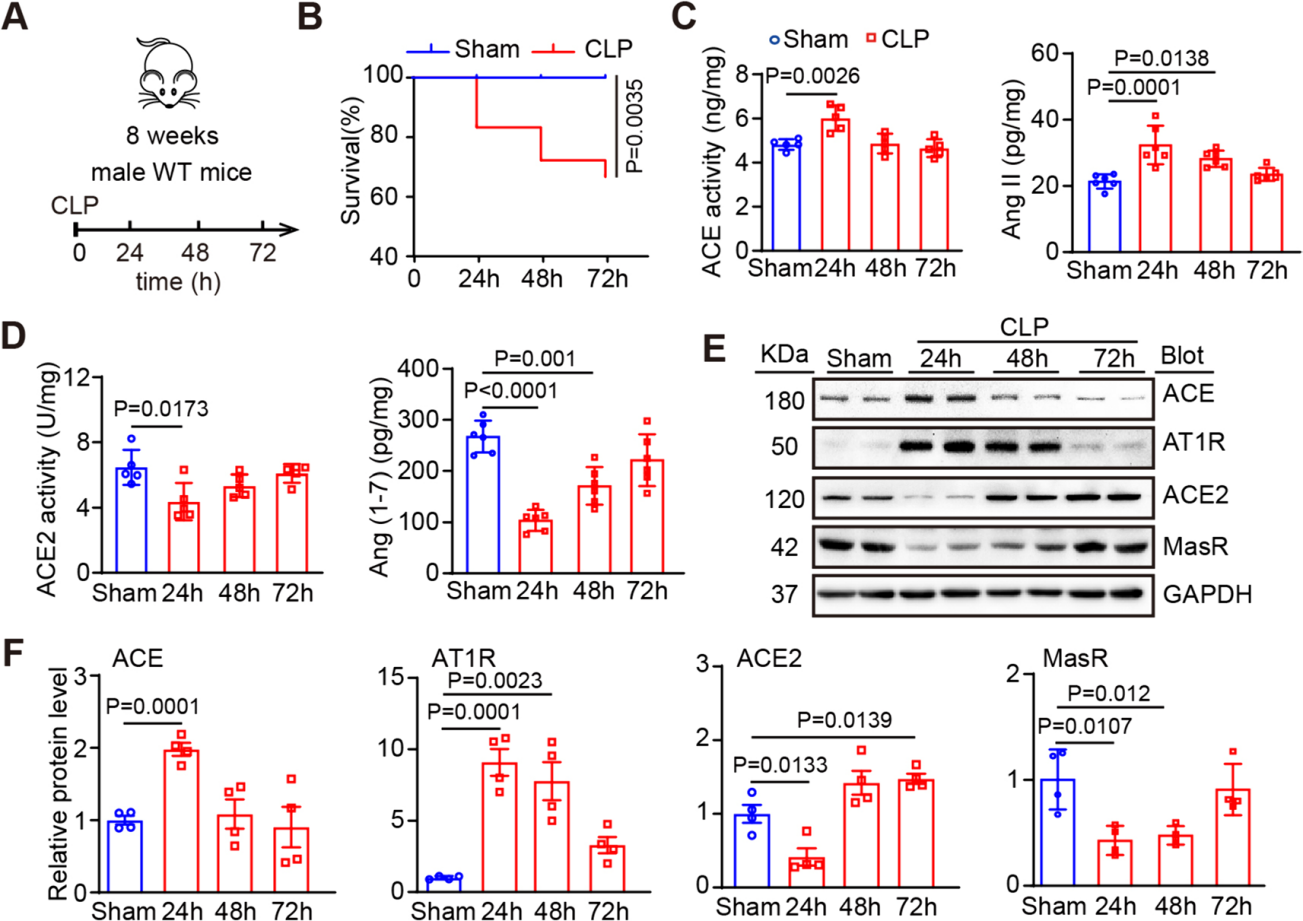
CLP disturbs the RAS balance in heart. **A** Experimental schematic diagram of WT mice subjected to CLP and observed for 24–72 h. **B** Effect of sham or CLP surgery on survival rate (*n* = 30). **C** Activity of ACE and level of Ang II in heart after CLP in WT mice (*n* = 6). **D** Activity of ACE2 and level of Ang (1–7) in the heart determined by ELISA (*n* = 4). **E** Western blot analysis of ACE, AT1R, ACE2, and MasR proteins in the hearts of mice after CLP. **F** Quantification of relative protein bands (*n* = 4). Data are presented as the mean ± SEM, and n represents the number of animals in each group

To explore the function of the ACE2 in SIC, we first established an SIC murine model via CLP (Fig. 1A). After 72 h, the survival of mice decreased with the duration after CLP surgery (Fig. 1B). Then, we determined the changes in the main components of the RAS in heart tissues 24–72 h following CLP operation. Our data showed that ACE activity and Ang II levels were significantly elevated, whereas ACE2 activity and Ang (1–7) level were markedly reduced in the heart at 24 or 48 h after CLP (Fig. 1C-D). Moreover, immunoblotting indicated the opposite tendencies of ACE-AT1R and ACE2-MasR axis in heart tissues, as showed by the upregulation of ACE and AT1R and downregulation of ACE2 and MasR expressions at 24 or 48 h after CLP (Fig. 1E-F). Together, these findings suggest that sepsis causes RAS imbalance by inhibiting ACE2 and activating ACE, thereby altering the levels of their catalytic products and the activity of downstream signaling pathways in the heart, which may be involved in the pathogenesis of SIC.

### Upregulation of ACE2 expression in mice attenuates CLP-induced cardiac dysfunction, oxidative stress and M1 macrophage polarization

**Fig. 2:**
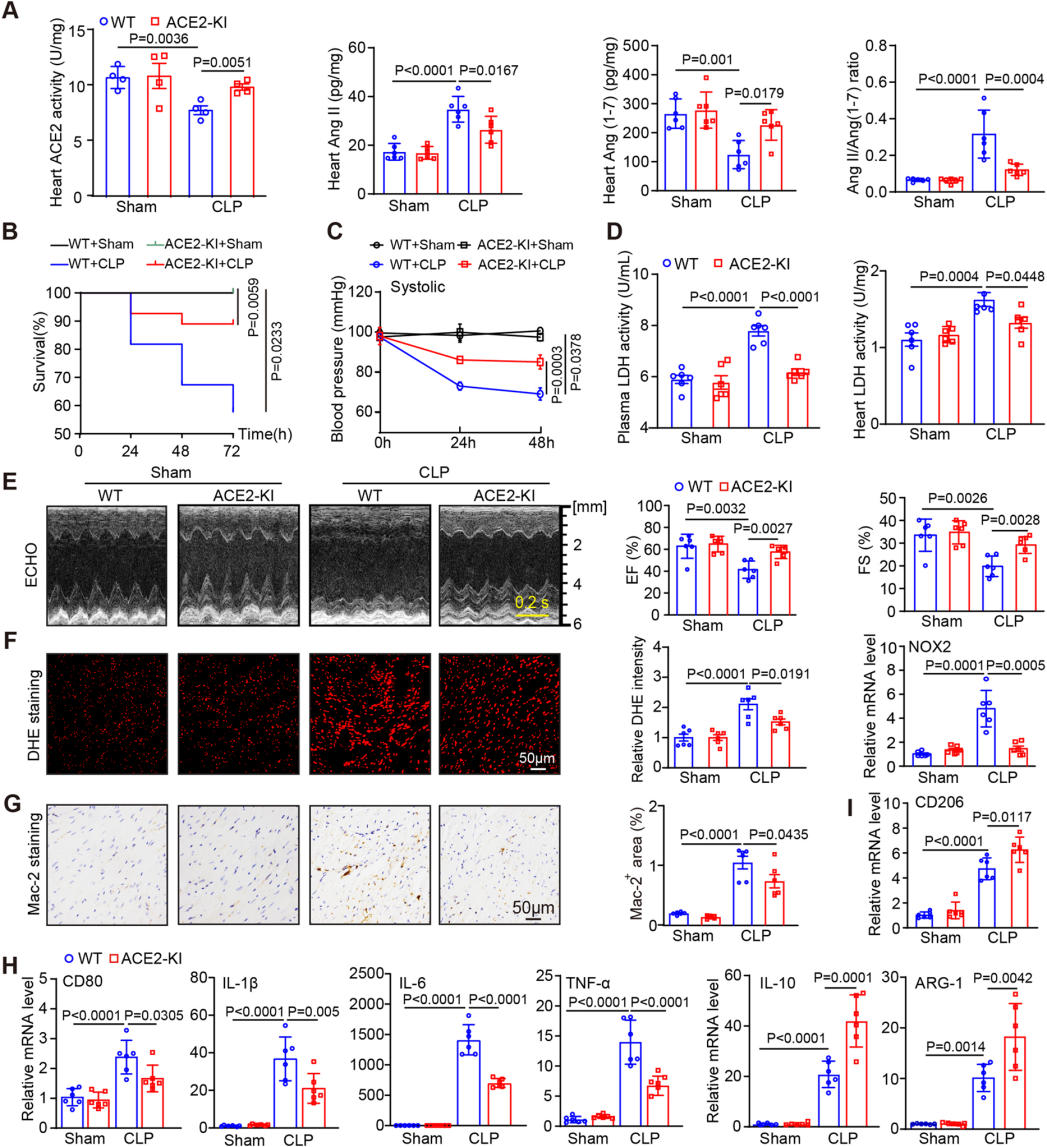
Overexpression of ACE2 attenuated myocardial injury, oxidative stress and M1 macrophage polarization induced by CLP. **A** ELISA analysis of activity of ACE2 and levels of Ang II and Ang (1–7) in the heart in WT or ACE2-KI mice after sham or CLP and Ang II to Ang (1–7) ratio (*n* = 4–6). **B** Survival rate of each group from 24 to 74 h (*n* = 20). **C** Measurement of systolic blood pressure (SBP) in each group by noninvasive tail-cuff method (n = 6). **D** Analysis of LDH levels in plasma and heart tissues in each group (*n* = 6). **E** M-mode echocardiography of the LV chamber (left) and measurement of the EF% and FS% (right, *n* = 6). **F** Dihydroethidium (DHE) staining of heart sections with quantification of the fluorescence intensity (left and middle) and qPCR analysis of the mRNA levels of NOX2 (right) in each group (*n* = 6). **G** Immunohistochemical staining of heart sections using Mac-2 antibody (left) with the percentage of Mac-2-positive areas (right, *n* = 6). **H-I** qPCR analysis of the mRNA levels of macrophage markers (CD80, IL-1β, IL-6, TNF-α, CD206, IL-10 and ARG-1) (*n* = 6). Data are presented as the mean ± SEM, and n represents the number of animals in each group

To examine whether increased ACE2 expression improves SIC, we generated ACE2-knockin (ACE2-KI) mice to overexpress ACE2 (Fig. S1A) and subjected them to CLP along with WT mice, followed by observation for 24–72 h. Our results revealed that there was no significant difference in body weight between WT and ACE2-KI at 8–10 weeks under normal condition (Fig. S1C). However, overexpression of ACE2 notably rebalanced the cardiac RAS, which was indicated by the reversal of reduced ACE2 activity by adjusting the Ang II/Ang (1–7) ratio through decreasing Ang II levels and increasing Ang (1–7) levels in the heart of CLP-treated mice compared to WT controls (Fig. 2A). Furthermore, the decreased survival rate and systolic blood pressure (SBP) induced by CLP were remarkably ameliorated in ACE2-KI mice in comparison with WT controls (Fig. 2B-C). Because the expression and activity of ACE2 were markedly decreased at 24 h after CLP (Fig. 1D-F), we assessed the function of ACE2 on the heart at this time point. Echocardiography indicated that upregulation of ACE2 protein in ACE2-KI mice conferred protection against CLP-induced cardiac damage (Fig. 2E). Specifically, there was a noticeable improvement in cardiac contractility, as evidenced by increased left ventricle EF% and FS%, coupled with a reduced LDH level (a marker of myocardial cytotoxicity) in both plasma and heart tissues at 24 h post-CLP compared to WT mice (Fig. 2D-E, Supplementary material, Table S2). Moreover, the level of plasma cardiac troponin I (cTnI) and the myocardial pathological scores in the ACE2-KI group were notably decreased than that observed in WT mice after CLP operation (Fig. S2A, S2C). The generation of ROS and inflammation can initiate oxidative damage and cell death (Cadenas 2018; Chouchani et al. 2014; Sun et al. 2021). Accordingly, immunostaining of heart tissues revealed that ACE2-KI significantly reduced superoxide production, NOX2 mRNA levels, and the percentage of Mac-2-positive macrophage infiltration in the heart compared to WT mice post-CLP operation (Fig. 2F-G). Furthermore, qPCR assays indicated that the CLP-induced rise in the mRNA levels of M1 macrophage markers (surface marker CD80 and cytokines IL-1β, IL-6 and TNF-α) and decline M2 macrophage markers (surface marker CD206 and cytokines IL-10 and ARG-1) in the heart were effectively countered in ACE2-KI mice exposed to CLP surgery (Fig. 2H-I). No statistically significant difference in these pathological features were observed between WT and ACE2-KI mice after the sham surgery. Collectively, these findings indicate that ACE2 inhibits macrophage M1 polarization and exerts anti-inflammatory effects during SIC, thus improving cardiac dysfunction induced by CLP via upregulating Ang (1–7) levels in the heart.

### Knockout of ACE2 aggravates CLP-induced cardiac dysfunction, oxidative stress and M1 macrophage polarization

**Fig. 3:**
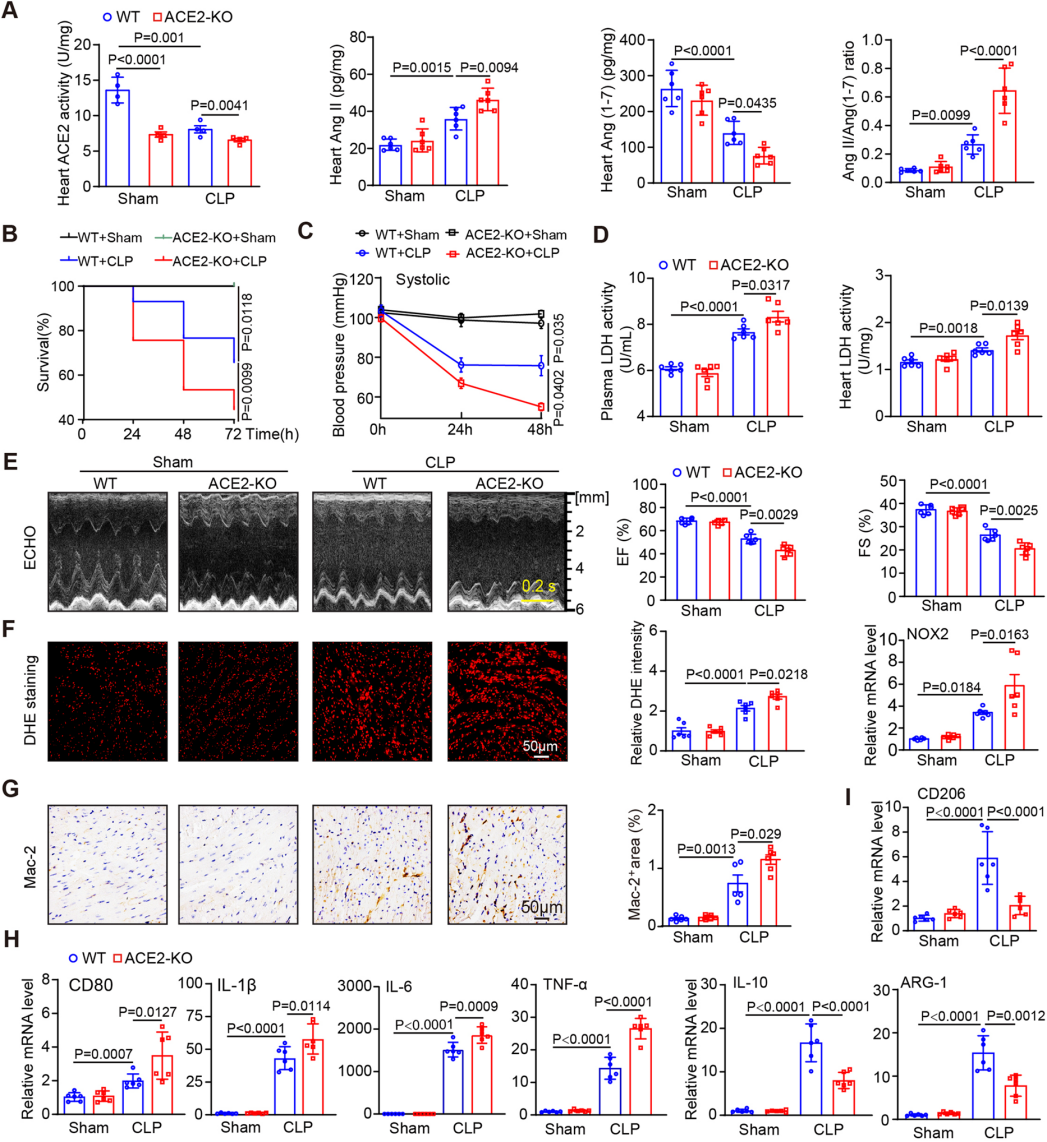
Deficiency of ACE2 aggravated myocardial injury, oxidative stress and M1 macrophage polarization induced by CLP. **A** ELISA analysis of ACE2 activity, levels of Ang II and Ang (1–7) and Ang II to Ang (1–7) ratio in the heart of WT or ACE2-KO mice following sham or CLP (*n* = 4–6). **B** Survival rate of each group from 24 to 74 h (*n* = 20). **C** Measurement of systolic blood pressure (SBP) in each group during 24–48 h by noninvasive tail-cuff method (*n* = 6). **D** Detection of LDH levels in plasma and heart tissues in each group (*n* = 6). **E** M-mode echocardiography of the LV chamber (left) and detection of the EF% and FS% (right, *n* = 6). **F** DHE staining of heart sections (left) and quantification of the fluorescence intensity (middle) and qPCR analysis of NOX2 mRNA levels (right, *n* = 6). **G** Immunohistochemical staining of heart sections with anti-Mac-2 antibody (left) and quantification of Mac-2-positive macrophages (right, *n* = 6). **H-I** qPCR analysis of the mRNA levels of CD80, IL-1β, IL-6, TNF-α, CD206, IL-10 and ARG-1 (*n* = 6). Data are presented as the mean ± SEM, and n represents the number of animals in each group

To validate the casual role of reduced ACE2 expression in SIC, we therefore produced global ACE2 knockout (ACE2-KO) mice (Fig. S1B) and then subjected them to sham or CLP for 24 h. Our results showed no significant body weight difference between WT and ACE2-KO mice at 8–10 weeks under basal conditions (Fig. S1C). However, the impaired of ACE2 activity of ACE2-KO mice resulted in a significant reduction of Ang (1–7) levels in the hearts, along with an increase in Ang II levels as well as Ang II/Ang (1–7) ratio in the heart of mice compared to WT controls (Fig. 3A). Furthermore, the decreased survival and SBP and increased cardiac dysfunction and damage (assessed by EF% and FS%, myocardial injury score, cTnI level, LDH activity), contents of superoxide (DHE staining), the mRNA levels of NOX2, and the percentage of Mac-2-positive macrophages in WT mice induced by CLP were greatly boosted in ACE2-KO mice (Fig. 3B-G, Supplementary material, Fig. S2B, S2D,Table S3). In addition, the mRNA expression of M1 polarization markers (CD80, IL-1β, IL-6, and TNF-α) were highly upregulated and the mRNA expression of and M2 polarization markers (CD206, IL-10 and ARG-1) were reduced in CLP-treated WT mice and these outcomes were remarkably aggravated in ACE2-KO animals treated with CLP (Fig. 3H-I). The parameters were comparable between WT and ACE2-KO mice following the sham surgery, with the exception of ACE2 activity. Therefore, these findings indicated that ACE2-KO shows enhanced susceptibility to cardiac dysfunction in sepsis by suppressing Ang (1–7) levels in the cardiac tissues.

### BM-derived ACE2-overexpressing cells attenuate CLP-induced cardiac dysfunction and inflammation

**Fig. 4:**
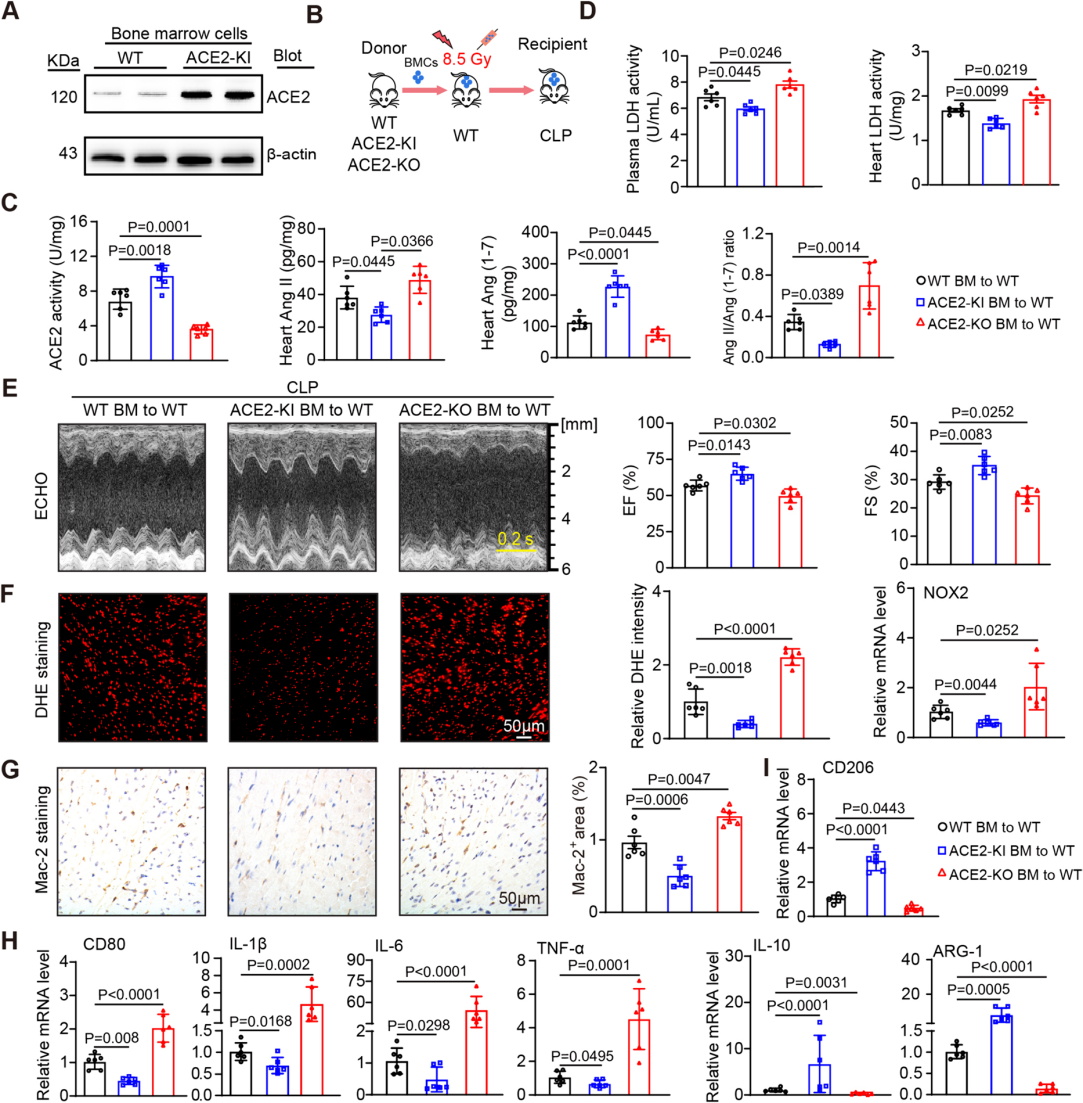
BM-derived cells overexpressing ACE2 prevent SIC. **A** Western blot experiment suggests increased expression of ACE2 in BM cells. **B** Experimental schematic diagram of BM transplantation. **C** Activity of ACE2 in the heart after CLP and levels of Ang II and Ang (1–7) and the ratio of the two determined by ELISA (*n* = 6). **D** Plasma (left) and heart tissue (right) LDH levels in each group (*n* = 6). **E** M-mode echocardiography of the LV chamber (left) and measurement of the EF% and FS% (right, *n* = 6). **F** DHE staining of heart sections with quantification of the fluorescence intensity (left and middle) and qPCR analysis of the NOX2 mRNA levels (right) (*n* = 6). **G** Immunohistochemical staining of heart sections using Mac-2 antibody (left) with the percentage of Mac-2-positive areas (right, *n* = 6). **H-I** qPCR analysis of the mRNA levels of CD80, IL-1β, IL-6, TNF-α, CD206, IL-10 and ARG-1 (*n* = 6). Data are presented as the mean ± SEM, and n represents the number of animals in each group

Interestingly, ACE2 was highly expressed in the BMDMs of ACE2-KI mice in comparison to WT animals (Fig. 4A), suggesting a crucial role for myeloid ACE2 in SIC. To directly determine whether ACE2 on myeloid cells is involved in the protection of SIC, we performed immunofluorescence staining on heart sections, which showed that ACE2 was colocalized with F4/80^+^ macrophages in cardiac tissues (Fig. S3). Then, we created various chimeric mice by BM transplantation for 8 weeks and then subjected them to CLP for 24 h (Fig. 4B). WT mice reconstituted with ACE2-KI BM showed a notable rise in ACE2 activity and a decline in the Ang II/Ang (1–7) ratio compared to WT mice reconstituted with WT BM, due to higher levels of Ang (1–7) and lower levels of Ang II during SIC (Fig. 4C). Meanwhile, reduced LDH levels in plasma and heart tissue, coupled with improved cardiac contractility (evidenced by the EF% and FS%), highlighted the protective role of ACE2-KI BM cells after CLP (Fig. 4D-E, Supplementary material, Table S4). Furthermore, WT mice reconstituted with ACE2-KI BM cells showed reduced ROS production and the number of Mac-2 positive macrophage infiltration, the mRNA levels of NOX2 and M1 markers (CD80, IL-1β, IL-6, and TNF-α) and elevated mRNA expression of M2 markers (CD206, IL-10 and ARG-1) compared to WT mice with WT BMCs (Fig. 4F-I). Conversely, the reconstitution of WT mice with ACE2-KO BM cells fully aggravated the above parameters and pathological changes in the heart induced by CLP (Fig. 4C-I). These findings suggest that myeloid overexpression of ACE2 predominantly mitigates cardiac dysfunction and inflammatory response induced by CLP.

### ACE2 overexpression suppresses NF-κB and STAT1 pathways, inhibits the polarization of M1 macrophages and the proinflammatory response induced by LPS through the activation of the Ang1-7-MasR axis

**Fig. 5:**
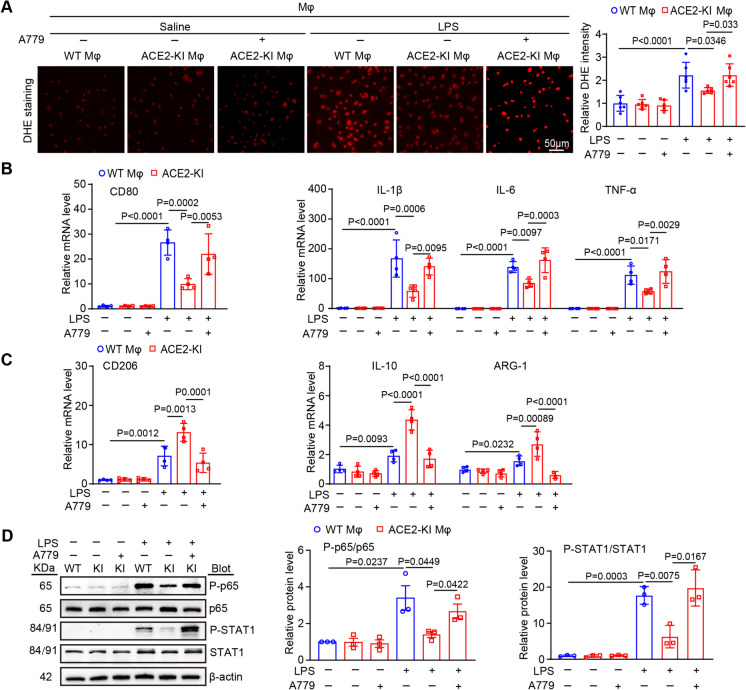
ACE2 overexpression mitigates macrophages polarization and hyperinflammatory response by inhibiting NF-κB and STAT1 pathways through activating the Ang (1–7)-MasR axis. BMDMs from WT or ACE2-KI mice were pretreated with the MasR antagonist A779 (10^–5^ Mol/L) for 1 h and then stimulated by saline or LPS (1 ng/μL) for another 24 h.** A** DHE staining of BMDMs (left) with quantification of the ROS fluorescence intensity (right, *n* = 3). **B-C** qPCR analysis of the mRNA levels of CD80, IL-1β, IL-6, TNF-α, CD206, IL-10 and ARG-1 in BMDMs of each group (*n* = 4). **D** Western blot analysis of P-p65 and P-STAT1 (left) in BMDMs with quantification of the relative protein bands (right, *n* = 3). Data are presented as the mean ± SEM, and n represents the number of independent experiments in each group

Given ACE2 is able to regulate macrophage polarization *in vivo*, we then analyze whether ACE2 overexpression inhibit macrophage M1 polarization via Ang (1–7)-MasR pathway *in vitro*. BMDMs were isolated from WT and ACE2-KI mice and pretreated with the MasR-specific antagonist A779 (10^–5^ mol/L) for 1 h, and then exposed them to LPS (1 ng/μL) for 24 h. DHE staining and qPCR analysis indicated that LPS dramatically enhanced the production of ROS and the mRNA levels of CD80, IL-1β, IL-6, and TNF-α but attenuated the mRNA levels of CD206, IL-10 and ARG-1 in BMDMs from WT mice, and these effects were markedly reversed in BMDMs from ACE2-KI mice (Fig. 5A-C). In contrast, A779 treatment markedly counteracted the preventive effects of ACE2-KI against LPS (Fig. 5A-C). These observations imply that ACE2 limits macrophage M1 differentiation via activating Ang (1–7)-MasR signal. To further assess the function of ACE2 on M1 macrophage proinflammatory response, we quantified the protein levels of key transcription factors (NF-κB and STAT1), to assess the role of them in the differentiation of M1 macrophages and the release of cytokines (Lawrence and Natoli 2011; Chávez-Galán et al. 2015). Immunoblotting results revealed an upregulation of phosphorylated P-p65 and P-STAT1 proteins in WT BMDMs after LPS stimulation, which was mitigated in ACE2-KI BMDMs (Fig. 5D). Conversely, A779 administration reversed the activation of NF-κB and STAT1 signals (Fig. 5D). No statistical differences in these parameters were obtained among these groups (Fig. 5A-D). Collectively, this evidence indicates that ACE2 modulates macrophage polarization and generation of pro-inflammatory cytokines by activating the Ang (1–7)-MasR signaling, leading to inhibition of NF-κB and STAT1 pathways within macrophages.

### ACE2 overexpression on macrophages alleviated cardiomyocyte ROS production and apoptosis

**Fig. 6:**
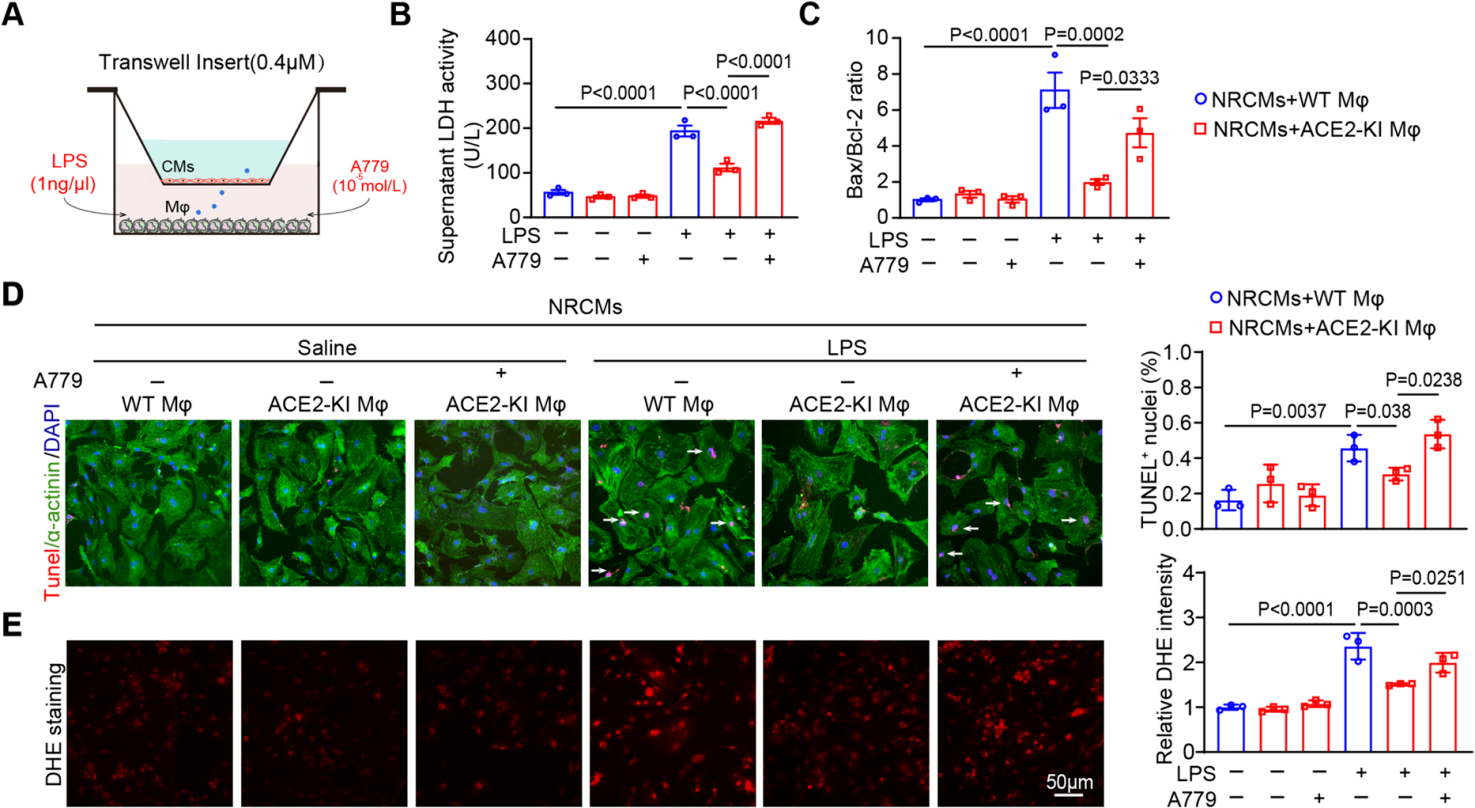
ACE2 overexpression on macrophages mitigates cardiomyocyte injury through activating the Ang (1–7)-MasR axis. **A** Experimental schematic diagram of co-culture BMDMs and NRCMs. **B** Measurement of LDH activity in the supernatant of NRCMs in each group (*n* = 3). **C** The ratio of Bax to Bcl-2 at mRNA level in NRCMs (*n* = 3). **D** Immunostaining of heart sections with TUNEL (red), α-actinin (green) and DAPI (blue) (left), and quantification of TUNEL-positive nuclei (right, *n* = 3). **E** DHE staining of NRCMs (left) with quantification of the ROS fluorescence intensity (right, *n* = 3). Data are presented as the mean ± SEM, and n represents the number of independent experiments in each group

**Fig. 7:**
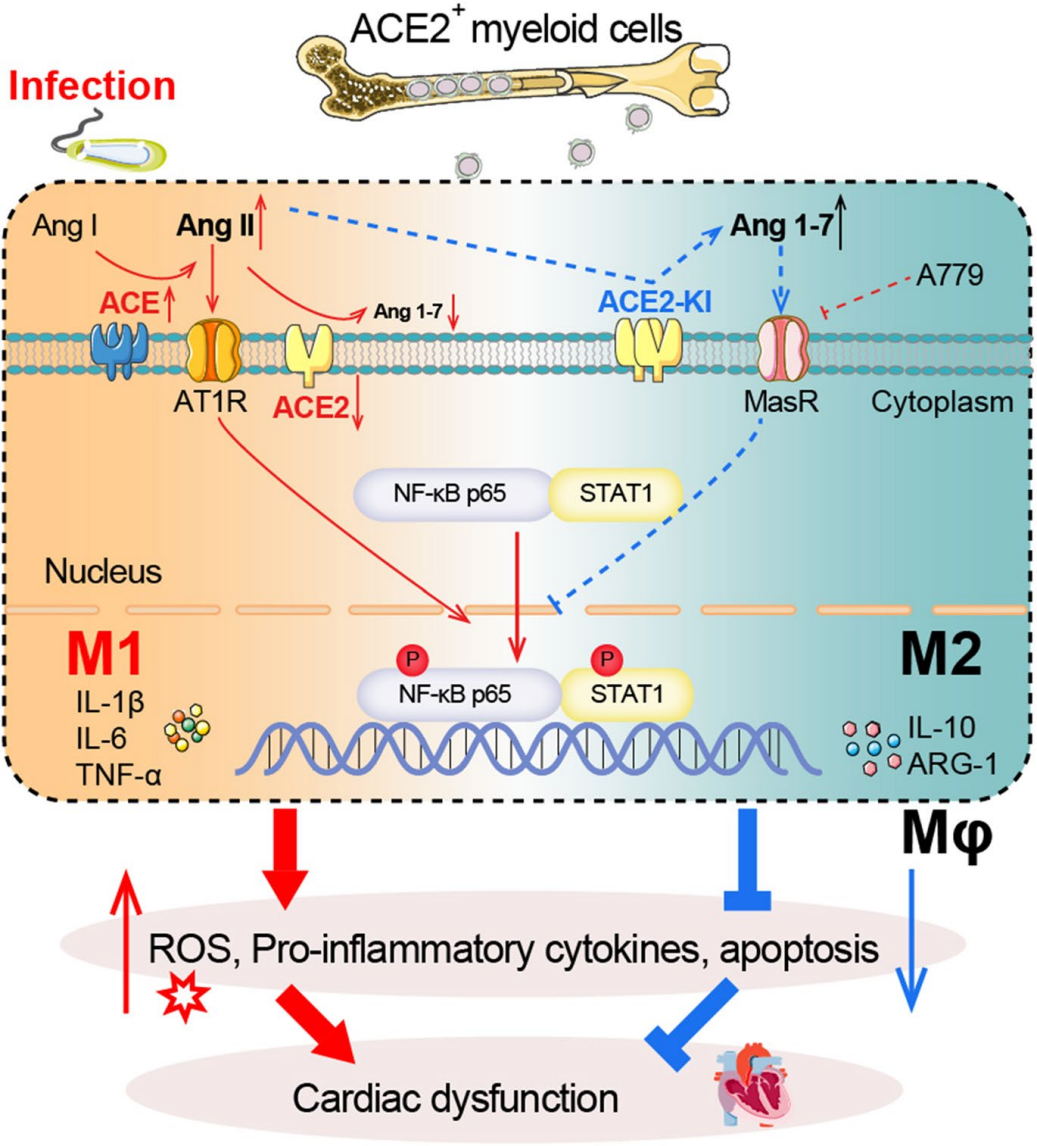
Mechanistic diagram illustrating the role of ACE2 in SIC. Sepsis reduces ACE2 expression in myeloid cells, leading to enhanced activation of the ACE-Ang II-AT1R axis and subsequent triggering of the NF-κB and STAT1 signaling pathways. This activation prompts macrophage polarization toward M1 phenotype, triggering the production of pro-inflammatory cytokines and ROS, promoting cardiomyocyte apoptosis, ultimately causing cardiac injury. Conversely, ACE2 overexpression in myeloid cells counteracts these effects by converting Ang II to Ang (1-7), which binds to MasR, inhibiting inflammation and ROS production. Consequently, this restoration of balance in the renin-angiotensin system promotes cardiac recovery. These findings underscore the potential of enhancing ACE2 activity or expression as a promising therapeutic strategy for the prevention and treatment of sepsis-induced cardiac dysfunction

Based on the above findings, experiments in the co-culture of BMDMs and NRCMs were conducted to substantiate whether ACE2’s regulation of macrophage polarization effectively mitigates cardiomyocyte damage (Fig. 6A). The LPS-induced M1 macrophage polarization elevated LDH levels, Bax/Bcl-2 mRNA ratio, the number of TUNEL-positive nuclei and the ROS production (DHE staining) in NRCMs incubated with WT BMDMs, and these effects were noticeably reduced when NRCMs were incubated with ACE2-KI BMDMs (Fig. 6B-E). However, treatment with MasR antagonist A779 effectively countered the ACE2-KI-mediated defense in BMDMs against the cardiomyocyte damage induced by LPS, including LDH activity, ROS elevation and cardiomyocyte apoptosis. (Fig. 6B-E). There were no statistical differences in these parameters among these groups (Fig. 6B-E). Taken together, these results demonstrated that ACE2 overexpression suppresses macrophage-induced cardiomyocyte ROS production and apoptosis via activating the Ang (1–7)-MasR axis (Fig. 7).

## Discussion

In this study, we initially confirmed ACE2 plays pivotal role in the development of murine SIC. Decreased ACE2 activity and Ang (1–7) contents were observed in the heart tissues after CLP surgery, implicating their involvement in sepsis-related pathophysiological processes. ACE2 overexpression significantly boosts the generation of Ang (1–7) and activation of MasR and subsequently inhibition of the NF-κB and STAT1 pathways. Thess effects subsequently mitigate the polarization of M1 macrophages and curtails the proinflammatory response and superoxide production, ultimately resulting in the amelioration of cardiac dysfunction during sepsis. Therefore, these results demonstrate that elevating ACE2 activity may present a promising therapeutic avenue for sepsis and SIC in patients.

The main function of ACE2 is able to transform Ang II to Ang (1–7), which initiates protective processes such as vasodilation and processes against proliferation and fibrosis by binding to its G-protein-coupled receptor (MasR). The deficiency of MasR could enhance the expression of pro-inflammation gene and promote macrophage infiltration and migration as well as polarization of M1 macrophage and Th1 cells (Hammer et al. 2016). In contrast, Ang II is produced through the enzymatic transformation of Ang I by ACE, binding to AT1R and consequently inducing vasoconstriction, proliferation, and fibrosis (Chamsi-Pasha et al. 2014). Underscoring the importance of the balance between the ACE2-Ang (1–7)-MasR and ACE-Ang II-AT1 axes (Meng et al. 2014), our research emphasizes a close correlation between SIC and local RAS homeostasis. During sepsis, we observed a decline in the activity of ACE2 and levels of Ang (1–7)-MasR, accompanied by an increase in the activity of ACE and the levels of Ang II-AT1R (Fig. 1C-F). ACE2 exhibits extensive expression throughout various tissues, including those of the heart, kidneys and testes. Within the cardiac context, ACE2 is distributed among cardiomyocytes, macrophages endothelial cells and smooth muscle cells (Iwata et al. 2011). Accumulating evidence emphasizes the preserving effects of ACE2 in cardiovascular diseases. Elevating ACE2 function and Ang (1–7) concentrations are beneficial for preventing and treat cardiac remodeling and heart failure (Iwata et al. 2011; Patel et al. 2017). Loss of ACE2 aggravates LV remodeling induced by myocardial infarction, while ACE2 overexpression or treatment of Ang (1–7) inhibits superoxide production and severe cardiac injury (Chen et al. 2023). A prospective cohort study demonstrated that recombinant human ACE2 (rhACE2) efficiently transformed Ang II to Ang (1–7), providing a potential novel treatment for heart failure (Basu et al. 2017; Patel et al. 2017). Zhong’s study revealed that ACE2 inhibits pathological hypertrophy, fibrosis and cardiac injury (Zhong et al. 2010). rhACE2 exerts a protective effect of sepsis-induced cardiomyopathy in mice through the enhancement of Ang II conversion to Ang (1–7) (Patel et al. 2017). A recent study showed that LPS treatment significantly reduced ACE2-Ang (1–7)-MasR axis protein levels in the cardiomyocytes and heart tissue in mice (Xu et al. 2021). Consistent with these findings, our study demonstrated that SIC alters the heart’s local RAS in mice by increasing the ACE-Ang II-AT1R axis and reducing the ACE2-Ang (1–7)-MasR axis. ACE2 overexpression alleviated CLP-induced EF% decline, ROS production and inflammation via activating the Ang (1–7)-MasR pathway, while loss of ACE2 aggravated the above dysfunction.

ACE2 exists in two forms: soluble ACE2 (sACE2) and membrane-bound ACE2, with the latter primarily expressed on epithelial cells and serving as the main receptor for SARS-CoV-2 virus. The extracellular portion of membrane-bound ACE2 can be shed into plasma, contributing to the infection process (Wang et al. 2023). Moreover, the recombinant spike protein of the SARS coronavirus alone reduces ACE2 expression on the cell surface *in vitro*, and mice administered with the spike protein showed decreased ACE2 expression and upregulated levels of Ang II (Kuba et al. 2005). This evidence imply that the virus may interact with the membrane bound ACE2, potentially cleaving it and subsequently releasing it into the bloodstream, thereby compromising local tissue function. In our study, decreased ACE2 activity in the heart was observed after CLP operation, suggesting that ACE2 is likely cleaved and released into the bloodstream. This leads to decreased levels and functionality of Ang (1–7), ultimately compromising its protective effect on the heart (Fig. 1D).

Sepsis, characterized by dysregulated host response to infection, leads to significant organ dysfunction, including cardiac complications (Cecconi et al. 2018; Gomez and Kellum 2016). Elevated production of ROS activates NF-κB signaling, leading to heightened release of proinflammatory cytokines (Chen et al. 2023). In the early stage of sepsis, monocytes/macrophages are rapidly recruited and release many inflammatory cytokines (e.g. IL-1β, IFN-γ, IL-6, TNF-α, and IL-10); subsequently, chemokines are released and acted on involved organs (Cavaillon and Adib-Conquy 2005). Some studies reported that LPS or IFN-γ can trigger the M1 macrophage polarization and accelerate inflammation and oxidative stress via the secretion of proinflammatory cytokines and superoxide, in turn impairing target organs. Macrophages that are influenced by IL-4 or IL-10 are designated anti-inflammatory M2 macrophages, which exert opposite effects against inflammation and oxidative stress and promote tissue repair (DeBerge et al. 2019; Lawrence and Natoli 2011). The pivotal role of macrophages in cardiovascular diseases has been demonstrated. Macrophages recognize polarized signals such as infection, hormones, and hypoxia to polarize to M1 type, releasing a large number of proinflammatory factors to damage cardiac function (DeBerge et al. 2019; Mouton et al. 2020).

Among the ingredients of the RAS, the ACE-Ang II-AT1 axis exhibits proinflammatory and pro-oxidative effects, while the ACE2-Ang (1–7)-MasR axes exhibits opposite outcomes (Li et al. 2022; Wang et al. 2012). Indeed, the ACE2 activator diminazene aceturate markedly decreases the influx of infiltrating inflammatory cells and mitigates endotoxin-induced uveitis in mice (Qiu et al. 2014). Thus, these markers are helpful for identifying infections before clinical symptoms appear (Karki et al. 2021; Pierrakos et al. 2020). Ang (1–7) and its decarboxylation product, alamandine, attenuate sepsis-associated cardiac dysfunction by decreasing the inflammatory reaction and cardiomyocyte apoptosis and inhibiting MAPK pathways (Li et al. 2018; Xu et al. 2021). Interestingly, the ACE2-Ang (1–7)-MasR axis attenuates M1 macrophage activity in septic mice via suppression of the NF-κB and MAPK pathways, which could be partly reversed with the administration of the MasR antagonist A779 (Pan et al. 2021). Our study firstly indicated that myeloid ACE2 contributes beneficially to mitigate SIC (Fig. 4). The transplantation of ACE2-KI mouse BM cells into WT mice alleviated the cardiac dysfunction, oxidative stress, inflammation and M1 polarization induced by CLP surgery through activation of the Ang (1–7)-MasR signal, while the transplantation of ACE2-KO mouse BM cells into WT mice counteracted the above effects (Fig. 4D-I). Furthermore, cell culture experiments *in vitro* demonstrated that ACE2 suppresses M1 macrophage polarization and hyperinflammatory response likely via Ang (1–7)-MasR-regulated inhibition of the NF-κB and STAT1 signals (Fig. 5A-D).

Limitations: Our study did not elucidate the mechanism behind the reduction of ACE2 in the heart 24 h after sepsis, nor did it include an examination of female mice. Based on the study, we found that ACE2 and Ang (1–7) were associated with SIC, but the causal relation between them was still uncertain. Future prospective studies are needed for explanation.

## Conclusion

Our study highlights the pivotal role of ACE2 in regulating macrophage polarization to alleviate SIC. The modulation of ACE2 activity and its downstream effects on Ang (1–7) production and MasR activation offer a novel avenue for the development of targeted therapies against SIC and potentially other sepsis-related cardiac complications. However, it is important to note that further investigations are needed to explore the precise mechanisms through which ACE2 influences macrophage polarization and to assess the safety and efficacy of ACE2-based interventions in clinical settings.

## Supplementary Information

Below is the link to the electronic supplementary material.

Supplementary file1 (DOCX 2018 KB)

## Data Availability

No datasets were generated or analysed during the current study.
